# Effects of repetitive gentle handling of male C57BL/6NCrl mice on comparative behavioural test results

**DOI:** 10.1038/s41598-020-60530-4

**Published:** 2020-02-26

**Authors:** Hiroshi Ueno, Yu Takahashi, Shunsuke Suemitsu, Shinji Murakami, Naoya Kitamura, Kenta Wani, Yosuke Matsumoto, Motoi Okamoto, Takeshi Ishihara

**Affiliations:** 1 Department of Medical Technology, Kawasaki University of Medical Welfare, Okayama, 701-0193 Japan; 2 Department of Psychiatry, Kawasaki Medical School, Okayama, 701-0192 Japan; 3 Department of Neuropsychiatry, Graduate School of Medicine, Dentistry and Pharmaceutical Sciences, Okayama University, Okayama, 700-8558 Japan; 4 Department of Medical Technology, Graduate School of Health Sciences, Okayama University, Okayama, 700-8558 Japan

**Keywords:** Cognitive neuroscience, Emotion, Learning and memory

## Abstract

Mice are the most commonly used laboratory animals for studying diseases, behaviour, and pharmacology. Behavioural experiment battery aids in evaluating abnormal behaviour in mice. During behavioural experiments, mice frequently experience human contact. However, the effects of repeated handling on mice behaviour remains unclear. To minimise mice stress, methods of moving mice using transparent tunnels or cups have been recommended but are impractical in behavioural tests. To investigate these effects, we used a behavioural test battery to assess differences between mice accustomed to the experimenter’s handling versus control mice. Repeatedly handled mice gained slightly more weight than control mice. In behavioural tests, repeatedly handled mice showed improved spatial cognition in the Y-maze test and reduced anxiety-like behaviour in the elevated plus-maze test. However, there was no change in anxiety-like behaviour in the light/dark transition test or open-field test. Grip strength, rotarod, sociability, tail suspension, Porsolt forced swim, and passive avoidance tests revealed no significant differences between repeatedly handled and control mice. Our findings demonstrated that mice repeatedly handled by the experimenter before behavioural tests showed reduced anxiety about high altitudes and improved spatial cognition, suggesting that repeated contact can affect the results of some behavioural tests.

## Introduction

Mice have been the most widely used laboratory animals for disease, behaviour, and pharmacology studies over the past century^1^. To successfully transfer the results obtained in mice to human studies, it is necessary to determine appropriate treatment and handling methods for experimental mice since handling of normal mice may affect the obtained experimental results.

Laboratory mice spend most of their lives in home cages before being used in experimental procedures. Environmental factors in the home cage play an important role in the health of these animals. The assessment of subjective animal welfare components relies primarily on physiological^2,3^ and behavioural^4–6^ measurements. Environmental conditions in animal facilities can affect the results of tests that measure natural behaviour in particular^7^. A factor often overlooked is how researchers handle animals. Lack of consideration for handling techniques can impede reproducibility and cast doubt on the relevance of otherwise valuable scientific research. Care and handling are integral parts of an animal’s daily life^8,9^, and handling is the most common procedure experienced by laboratory mice because it is necessary for routine (e.g. cage cleaning and breeding) and research (e.g. injections and blood sampling) procedures. For practical reasons, animals are usually subjected to stress between periods of inactivity during the light cycle^10^. To improve the reproducibility of animal studies, we need to consider all stressors that may influence the experimental animals, and handling is one of them. Insufficient description of this process can cause experimenter errors and biases^11^.

Often, laboratory mice are evaluated for depression, aggression, activity, and anxiety-like behaviour through a series of behavioural tests to analyse the effects of drugs or other stimuli^12^. Behavioural tests in mice are important tests that have been performed since a long time and are widely used worldwide^13–16^. In particular, conducting a behavioural test battery is extremely important for detecting abnormal behaviour in mice^17–19^. In many behavioural tests, the experimenter frequently needs to come into contact with the mouse. Thus, differences in handling techniques can greatly affect the results of behavioural tests. However, it is often unclear how a laboratory or researcher handles the mice^20–23^. Consequently, the results of behavioural tests often differ among researchers. In behavioural tests, it is important to understand the types of these tests susceptible to background stress and how to minimize its influence.

In many experiments, including behavioural tests, the most common method used to capture and carry mice is to pick their tail at its base between the thumb and index finger. This is specified in standardized protocols for the handling of experimental mice^24–26^. However, lifting the mouse by its tail is aversive to the mouse, causing stress and anxiety. Studies on behavioural phenotypes in mice have identified stress handling as one of the most likely causes of failure to replicate phenotypes within and between experiments^27^.

In recent years, “tunnelling” and “cupping” have been recommended to eliminate the drawbacks of tail handling^28,29^. These studies used tools (cups and transparent tunnels) to move the mouse to a specific location, and their authors describe that these methods reduce the effect on the anxiety levels in mice. Using tunnels or cups has been recommended by additional studies as a method to reduce anxiety-like behaviour in mice if their handling is unavoidable^30–32^. In fact, handling tunnels and cups minimize human contact with animals, but it is impractical to perform all experimental work solely with tunnels and cups. It is extremely difficult to place mice in or remove them from various behavioural experimental devices and inject drugs into or collect blood from these mice without grabbing their tails.

Therefore, in this study, mice were adapted to the hands of an experimenter before the behavioural test. Once a day for three weeks, we grabbed mice by their tails, lifted them, and let them rest in the hands of an experimenter for 30 s. It was speculated that mice would learn that these procedures are not harmful to them even if the experimenter grabbed their tails, thus reducing anxiety. In a following series of behavioural experiments, mice were moved by grabbing their tails. We clarified how mice that were familiar with the experimenter’s hand were affected in a series of behavioural test batteries. Reducing handling-induced anxiety in mice as a source of variation in behavioural tests may contribute to a reduction in the number of animals required for experiments. In addition to providing more robust scientific results, it is speculated that the use of appropriate handling techniques will enhance the well-being of mice used worldwide.

## Results

### Increased familiarity with the experimenter’s hand

**Figure 1:**
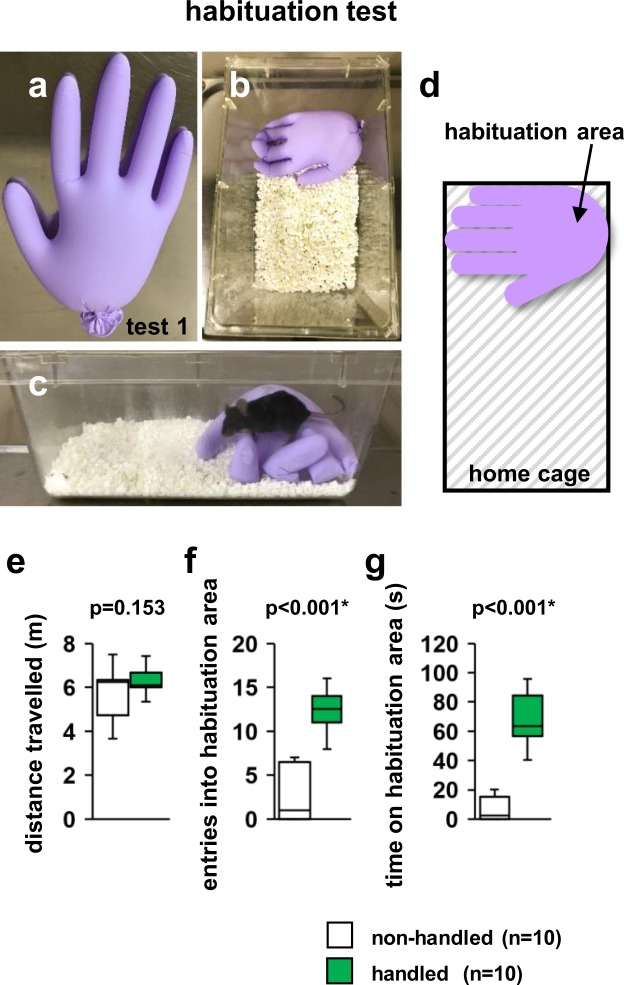
Effect of repeated handling on the performance in the habituation test. (**a**) Sample picture of a glove filled with water. (**b**) Sample picture depicting a new home cage with a glove. (**c**) Sample picture during the habituation test. (**d**) Schematic diagram of the habituation test. Total distance travelled (**e**), number of entries into the habituation area (**f**), and time spent in the habituation area (**g**). All data are presented as box plots. *, significant difference compared to control data (p < 0.05); p-values were calculated using the one-way ANOVA. (**e–g**) non-handled: n = 10, handled: n = 10.

In the habituation test, there were no significant differences in the total distance travelled between non-handled and handled mice (Fig. 1e, *F*_1,18_ = 2.222, p = 0.153).

The number of entries into the habituation area and time spent in this area were significantly increased in handled mice compared to those in non-handled mice (Fig. 1f, *F*_1,18_ = 51.200, p <0.001; Fig. 1g, *F*_1,18_ = 45.844, p <0.001).

### General characterization of handled mice

**Figure 2:**
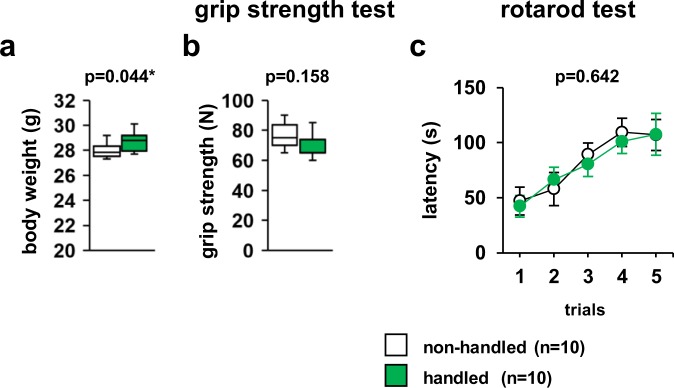
Effect of repeated handling of mice on their physical characteristics. Body weight (**a**), grip strength (**b**), and latency to fall in the rotarod test (**c**). All data are presented as box plots. *Significant difference compared to control data (p < 0.05); p-values were calculated using the one-way ANOVA (**a,b**) or two-way repeated-measures ANOVA (**c**). (**a–c**) non-handled: n = 10, handled: n = 10.

As shown in Fig. 2a, handled mice showed an increased body weight (*F*_1,18_ = 4.663, p = 0.044). There were no significant differences between non-handled and handled mice in terms of grip strength (Fig. 2b, *F*_1,18_ = 2.170, p = 0.157) and latency to fall in the rotarod test (Fig. 2c, *F*_4,72_ = 0.309, p = 0.642).

### Decreased anxiety-like behaviour of handled mice in the elevated plus-maze test

**Figure 3:**
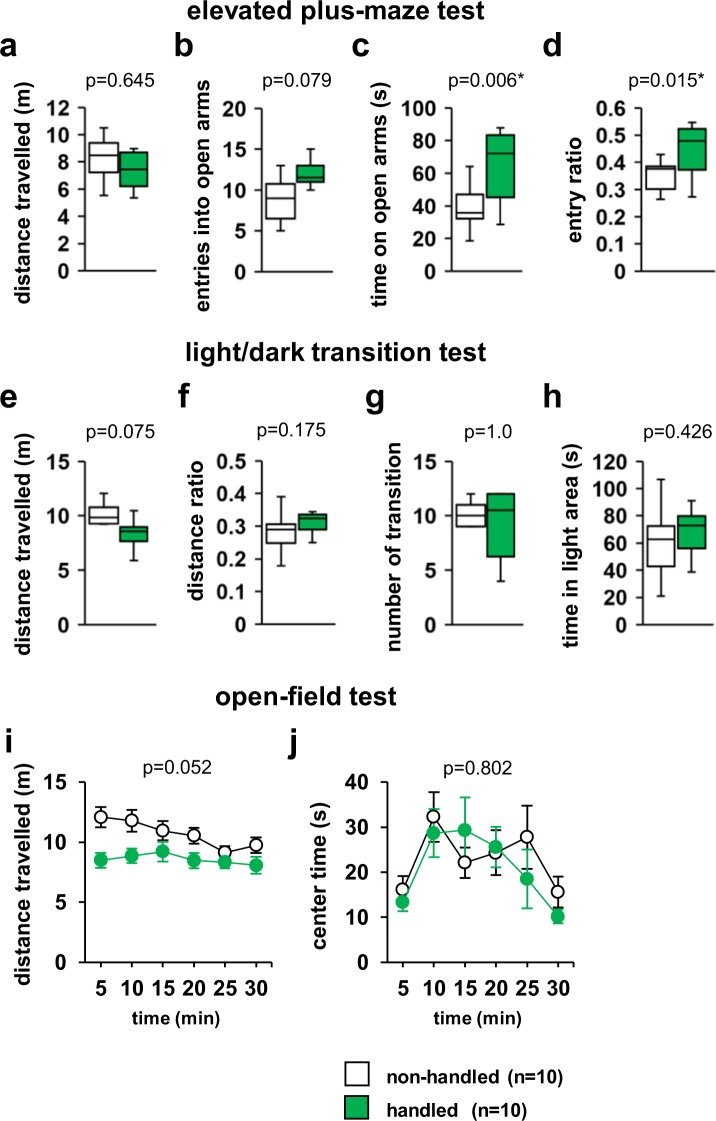
Effect of repeated handling on anxiety-like behaviour. Elevated plus-maze test: Total distance travelled (**a**), number of entries into open arms (**b**), and time spent in open arms (**c**), and ratio of entries into the open arms (**d**). Light/dark transition test: Total distance travelled (**e**), ratio of distance in the light compartment (**f**), number of light/dark transitions (**g**), and time spent in the light compartment (**h**). Open-field test: Distance travelled in each of the 5-min test periods (**i**) and time spent in the central area in each of the 5-min test periods (**j**). Data are presented as box plots (**a–h**) or the mean ± standard error (**i,j**). *significant difference compared to controls (p < 0.05); p-values were calculated using the one-way ANOVA (**a–h**) or two-way repeated-measures ANOVA (**i,j**). (**a–j**) non-handled: n = 10, handled: n = 10.

In the elevated plus-maze test, there were no significant differences between non-handled and handled mice in the total distance travelled (Fig. 3a, *F*_1,18_ = 0.219, p = 0.645) and total number of entries into open arms (Fig. 3b, *F*_1,18_ = 3.457, p = 0.079). However, handled mice exhibited a significantly increased time spent in the open arms (Fig. 3c, *F*_1,18_ = 9.381, p = 0.006) compared with non-handled mice. Additionally, handled mice exhibited a significantly increased ratio of entries into open arms (Fig. 3d, *F*_1,18_ = 7.102, p = 0.015) compared with non-handled mice.

### Unaltered light/dark transition in handled mice

In the light/dark transition test, no significant differences were observed in the total distance travelled (Fig. 3e, *F*_1,18_ = 3.547, p = 0.075), ratio of distance in the light compartment (Fig. 3f, *F*_1,18_ = 1.992, p = 0.175), number of transitions between light/dark compartments (Fig. 3g, *F*_1,18_ = 0.0, p = 1.0), and time spent in the light compartment (Fig. 3h, *F*_1,18_ = 0.662, p = 0.426) between non-handled and handled mice.

### Effects of handling in the open-field test

In the open-field test, no significant differences between non-handled and handled mice were detected regarding distances travelled (Fig. 3i, *F*_14,90_ = 2.295, p = 0.052) and centre time (Fig. 3j, *F*_14,90_ = 1.016, p = 0.802).

### Increased alternation percentage of handled mice in the Y-maze test

**Figure 4:**
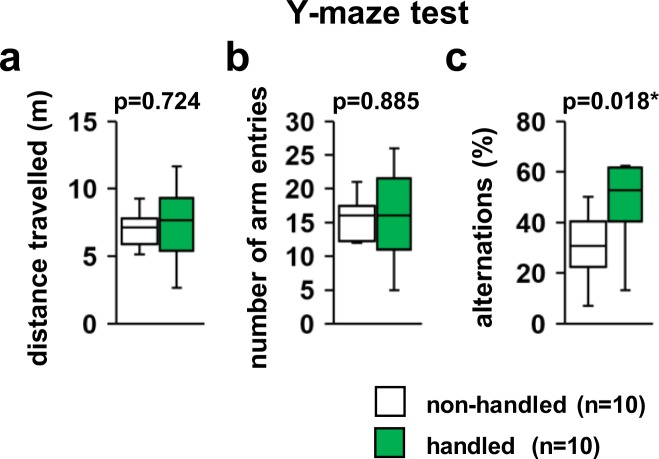
Effect of repeated handling on the performance of mice in the Y-maze test. The total distance travelled (**a**), total number of arm entries (**b**), and percentage of alternations (**c**) are depicted. All data are presented as box plots. *significant difference compared to controls (p < 0.05); p-values were calculated using the one-way ANOVA. (**a–c**) non-handled: n = 10, handled: n = 10.

In the Y-maze test, there were no significant differences between non-handled and handled mice in the total distance travelled (Fig. 4a, *F*_1,18_ = 0.128, p = 0.724) and number of arm entries (Fig. 4b, *F*_1,18_ = 0.021, p = 0.885). In contrast, the percentage of alternations in the total number of entries was significantly increased in handled mice (Fig. 4c, *F*_1,18_ = 6.726, p = 0.018) compared to non-handled mice.

### Social behaviour in handled mice

**Figure 5:**
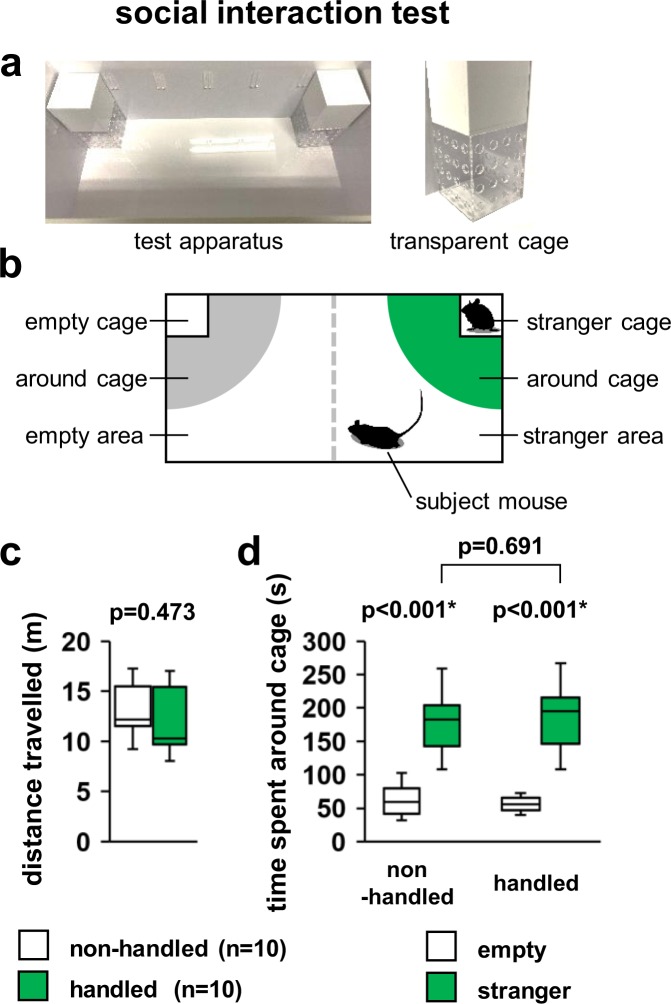
Effect of repeated handling on performance in the murine sociability test. Sample picture (**a**) and schematic diagram (**b**) of this test. Total distance travelled (**c**) and time spent near the cage (**d**) in the sociability test. Data are presented as box plots. *significant difference compared to controls (p < 0.05); p-values were calculated using the one-way ANOVA (**c**) or repeated-measures ANOVA (**d**). (**c,d**) non-handled: n = 10, handled: n = 10.

In the sociability test, there was no significant difference between non-handled and handled mice in the total distance travelled (Fig. 5c, *F*_1,18_ = 0.543, p = 0.473). Both non-handled and handled mice spent more time near the stranger-side cage than the empty-side cage (Fig. 5d; non-handled: *F*_1,14_ = 32.534, p < 0.001; handled: *F*_1,14_ = 40.258, p < 0.001). There was also no significant difference between non-handled and handled mice in the time spent near the stranger side (Fig. 5d, *F*_1,14_ = 0.164, p = 0.691).

### Normal immobility of handled mice in tests for depression-like behaviour

**Figure 6:**
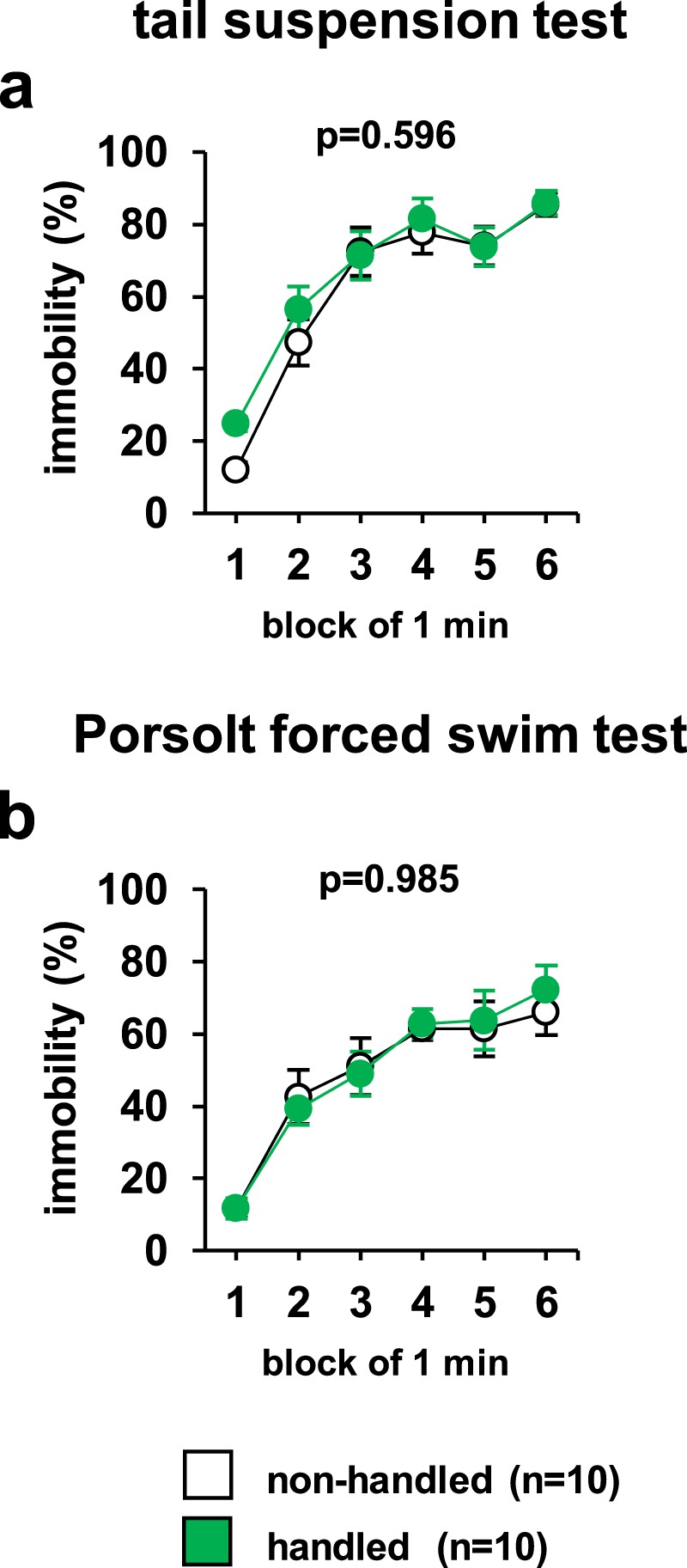
Effect of repeated handling on depressive-like behaviour. Graphs showing the proportion of time spent immobile in each 1-min period in the tail suspension test (**a**) or in the Porsolt forced swim test (**b**). Data are presented as means ± standard errors. *significant difference compared to controls (p < 0.05). p-values were calculated using the two-way repeated-measures ANOVA. (**a,b**) non-handled: n = 10, handled: n = 10.

In the tail suspension test, there was no significant difference between non-handled and handled mice regarding the percentage of time spent immobile (Fig. 6a, *F*_14,90_ = 0.714, p = 0.596). In the Porsolt forced swim test, there was no significant difference between non-handled and handled mice in the percentage of time spent immobile (Fig. 6b, *F*_14,90_ = 0.180, p = 0.985).

### Normal learning and memory of handled mice in the passive avoidance test

**Figure 7:**
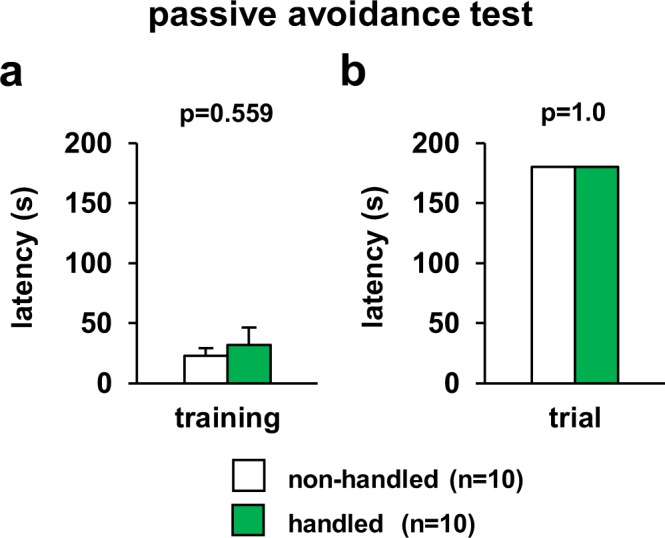
Effect of repeated handling on performance on the passive avoidance test. Graphs showing the escape latencies during the training session (**a**) and the retention test (**b**). In the retention test 24 h after training, the maximum latency was set as 180 s. Data are presented as means ± standard errors. *significant difference compared to controls (p < 0.05). p-values were calculated using the one-way ANOVA. (**a,b**) non-handled: n = 10, handled: n = 10.

In the step-through passive avoidance test, there was no significant difference between non-handled and handled mice in the latency to enter the dark compartment throughout the conditioning session (Fig. 7a, *F*_1,18_ = 0.353, p = 0.559). At 24 h after the conditioning, the step-through latency in handled mice was not lower than that in non-handled mice in the retention trials (Fig. 7b, *F*_1,18_ = 0.0, p = 1.0).

## Discussion

In this study, we demonstrated that mice exposed repeatedly to tool-free handling exhibited reduced anxiety about high altitudes in the elevated plus-maze test. Interestingly, repeatedly treated mice showed no significant differences in anxiety-like behaviour in the open-field test and the light/dark transition test compared to the non-handled control group. In addition, repeatedly treated mice were clearly accustomed to the hands of the experimenter but did not show significant differences in many other behavioural tests.

In the habituation test, repeatedly handled mice frequently stayed on water-filled gloves for long periods of time, whereas non-handled control mice hardly ever rested on gloves in this test, indicating that mice that were repeatedly handled were clearly accustomed to the experimenter’s hand and had no fear of the hand. In mice, the degree of aversion to tail-grabbing is unknown, but our study results indicate that there is no additional influence of the glove on the tail-grabbing procedure. This new test method may help non-handled mice become familiar with the experimenter’s hand. Further studies are needed to determine the effect of this method on behavioural changes.

Mice handled repeatedly showed a slight increase in body weight compared to control mice. The exact mechanism causing the weight increase is unknown. It has been reported that mice may have increased appetite due to chronic stress^33^. It is possible that mice that had been lifted by their tails once a day experienced chronic stress, leading to increased appetite in these mice. In recent years, it has been shown that the results of rodent pain responses differ depending on the sex of the experimenter^34^. In particular, exposure to male experimenters has been reported to cause rodent stress^34^. Our results suggest that although the handled mice were accustomed to the experimenter’s hands, they were not accustomed to being gripped by the experimenter or treated daily.

The elevated plus-maze test is a widely accepted method for examining the efficacy of anxiolytics^35–37^. Anxiety-like behaviour has also been measured in the elevated plus-maze test using model mice for neuropsychiatric disorders^38,39^. In the present study, repeatedly handled mice had significantly reduced anxiety-like behaviour in the elevated plus-maze test. However, repeatedly treated mice showed no decrease in anxiety-like behaviour in the open-field test and the light/dark transition test. Although there was no significant difference, mice handled repeatedly tended to move less than control mice. Furthermore, the distance travelled for both groups did not decrease over the 30-min test period, suggesting that they were not accustomed to the test environment. The open-field test examines anxiety-like behaviour in a larger space^40,41^, whereas the light-dark transition test is a behavioural experiment that measures anxiety in an illuminated space^42,43^. There are several types of anxiety-like behaviour, such as fear of elevated spaces, of illuminated spaces, or of vast, open spaces. Therefore, it is very important to conduct a series of behavioural tests. The results of this study suggest that mice handled repeatedly have reduced height-induced anxiety. Mice adapt to the handling by the experimenter^1^. Thus, repeatedly handled mice likely became accustomed to heights because they were in the hands of the experimenter 50 cm above the cage once a day. These results support the hypothesis that handling of mice prior to behavioural testing may alter their emotional state.

Anxiety can affect the performance of a mouse in certain types of experiments. Increased anxiety diminishes the attention and makes it difficult for mice to respond properly to the stimuli of interest. Tasks that require learning, memory, and problem solving (such as the frequently used T-maze) are even more difficult. In the present study, repeatedly handled mice showed improved spatial cognitive functions in the Y-maze test. This suggests that these mice may have reduced anxiety levels. To evaluate the extent of improvement in the spatial cognitive function, it is necessary to perform additional behavioural experiments such as the T-maze test. Conversely, the alteration ratio in the Y-maze test varies between 30% and 70% depending on the laboratory^20–23^. Thus the 30% alteration ratio in control mice observed in this study might have been low.

Anxiety and depression are thought to be relatively strongly correlated comorbidities in both humans and animals^44–46^. If anxiety levels are substantially reduced in repeatedly handled mice, we would expect depression-like behaviour to be similarly reduced. However, the outcomes of this study did not indicate a decrease in depression-like behaviour. It is unknown whether repeated handling does affect anxiety or whether a series of behavioural tests increase anxiety. The results may have differed if the tail suspension test and forced swimming test had been performed on the first day of behavioural tests. Porsolt forced swim test assumes that a second exposure trials measures “behavioural despair” and/or “learned helplessness”^47^. In this study, it was possible that there was a significant difference between the groups in the second exposure trials.

Social hierarchy is formed amongst male mice bred in the same cage^48^. Dominant mice exhibit higher locomotor and exploratory activities^49^. However, the effects of mice hierarchy on their health are largely unknown. The behavioural changes seen in the present study might also have been influenced by social hierarchy.

Recently, “tunnelling” and “cupping” have been recommended to eliminate the drawbacks of grabbing mice by their tails^28,29^. The use of tunnels and cups reduces anxiety in mice compared to standard tail handling^28,29^. However, the reduction of anxiety-like behaviour using the cup-based method has only been assessed using the elevated plus-maze test^28^. Open-field or light/dark transition tests that examine other anxiety-like behaviours have not been performed in these studies. Moreover, the authors recommend that the tunnels used should be transparent^28,29^. However, if the transparency is high, the mouse inside the tunnel can observe the environment and may lose its fear of high places. Considering these facts, it is highly probable that the recently recommended methods using cups and tunnels are making mice accustomed to high altitudes, and it is questionable whether they actually reduce various anxiety-like behaviours in mice. Furthermore, it has also been reported that mice undergoing tunnelling do not exhibit altered anxiety-like behaviour^32^. In this study, the mice used for comparison were held by their tails for 30 s every day by the experimenter to induce elevation-related anxiety. This corresponds to performing a repeated tail suspension test that induces high stress levels^50^. However, not many behavioural tests contain the task of holding a mouse by its tail for 30 s, and it is thus an unrealistic experimental paradigm. Present study also suggests the need to reconsider the efficacy of transport methods using cups and tunnels.

Handling-induced behavioural changes have been demonstrated in rats for many years. These effects have been well studied because rats have been favoured in experimental studies evaluating learning and memory. In recent years, attention has been focused on mouse behaviour due to increased interest in transgenic mice^51,52^. Mice have been shown to be sensitive to the type of handling and presence of researchers^29,53,54^. In the laboratory, handling of mice cannot be avoided. Reducing mouse anxiety due to handling as a source of variability in experimental studies contributes to a reduction in the number of animals required for experiments. In addition to providing more robust scientific results, it is speculated that proper selection of handling methods will enhance the well-being of mice used in laboratories worldwide.

This research demonstrated that there is no significant difference in the results of the open-field test, light/dark transition test, rotarod test, tail suspension test, Porsolt forced swim test, and passive avoidance test between repeatedly handled and non-handled mice. This also indicates that there is no need to adapt mice to the experimenter’s hand prior to conducting these behavioural tests.

## Conclusion

Repeatedly exposing the mice to the experimenter’s hand before conducting behavioural tests allows them to get accustomed to it and to reduce anxiety about high altitudes. However, this treatment does not affect other forms of anxiety such as fear of open or illuminated spaces. Furthermore, getting accustomed to the experimenters’ hands does not affect the results in most behavioural experiments. Frequent mice handling prior to conducting the behavioural test battery may influence anxiety-like behaviour tests at high altitudes. Our study also indicates that frequent mice handling in order to accustom the mice to the experimenter’s hands is not essential prior to conducting the behavioural test battery.

## Materials and Methods

### Animals

All animal experiments were performed in accordance with the U.S. National Institutes of Health (NIH) Guide for the Care and Use of Laboratory Animals (NIH Publication No. 80–23, revised in 1996) and were approved by the Committee for Animal Experiments at the Kawasaki Medical School’s Advanced Research Center. All efforts were made to minimise the number of animals used and to prevent avoidable discomfort. Male C57BL/6 N mice (age: 10 weeks) were purchased from Charles River Laboratories Japan (Kanagawa, Japan). Five mice were housed in a plastic cage (20 × 30 × 20 cm) with a stainless-steel lid, with food and water provided *ad libitum* under a 12-h light/dark cycle at 23 °C−26 °C, at a humidity of 20–50% with no enrichment structures in the cage. To habituate the mice to behavioural testing room conditions, we placed mouse cages in the laboratory for 1 h daily for 4 days.

### Experimental design

**Figure 8:**
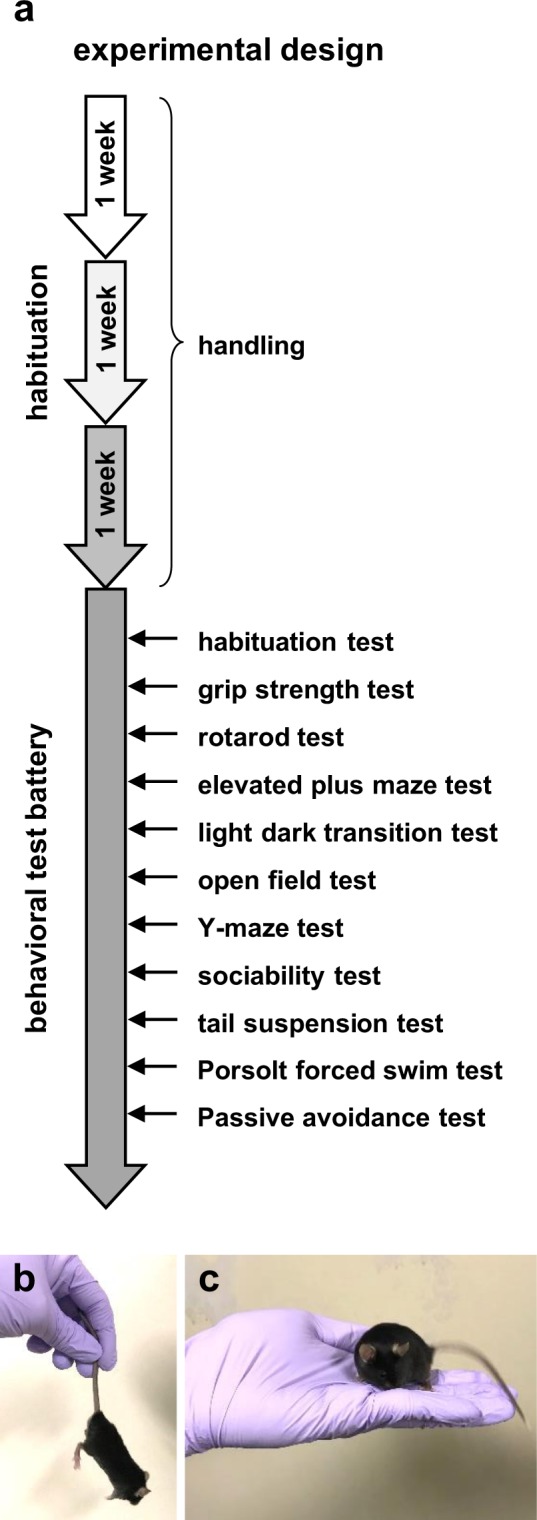
Experimental design. (**a**) Experimental time schedules. Animals in the repeated handling groups were subjected to handling once a day for three weeks. Afterwards, we performed the behavioural test battery. Mice were subjected to two behavioural tests per day. (**b**) Sample picture of grasping the mouse by its tail before transferring it on the hand of the experimenter. (**c**) Sample picture of a mouse in the experimenter’s hand.

The series of experiments were performed by a single male experimenter. Mice were randomly divided into two groups; one group was exposed to repeated handling (n = 10), and the other was not (n = 10). Over the course of 21 days, the mice were handled every day in the evening hours during the light cycle (13:00–17:00). Behavioural experiments began on the 22^nd^ day (Fig. 8a). All cages were moved to the behavioural testing room, whereas one group was exposed to handling. Handled mice were removed from their home cages with their tails grabbed (Fig. 8b) and placed on the palm of the experimenter for 30 s (Fig. 8c). The researcher’s hand was positioned 50 cm above the home cage. Mice were allowed to explore freely during the 30-s handling exposure period. After 30 s, the hand was placed in the home cage, and the mouse was able to jump from the hand into the cage. In contrast, mice in the control group was not handled by the researchers and were only touched by the researchers during cage changes. Handled and non-handled mice were contacted by the same researchers.

### Behavioural tests

All behavioural tests were conducted in behavioural testing rooms between 13:00 and 17:00 during the light phase of the light/dark cycle. Behavioural tests were performed in mice between the ages of 13 and 14 weeks. Each behavioural test was performed within at least 1 day of each other. Mice were tested in a random order. After the tests, the equipment was cleaned with 70% ethanol and super hypochlorous water to eliminate olfactory cues. The behavioural testing rooms were illuminated at 100-lux intensity.

### Habituation test

To determine whether the mice got accustomed to the researcher’s hands, we examined their willingness to explore gloves presented as an unfamiliar cue. A sealed glove was filled with water (Fig. 1a) and placed on the edge of a new home cage (20 × 30 × 20 cm) (Fig. 1b). The top of the water-filled glove was defined as the “habituation area” (Fig. 1c,d). Each mouse was placed outside this habituation area and was allowed to move freely for 6 min. The movements of these mice were recorded on video, and the number of habitation area entries, distance travelled (m), and time spent in the habituation area (s) were analysed using a video tracking software (ANY-MAZE, Stoelting Co., Wood Dale, IL, USA).

### Neuromuscular strength evaluation

Neuromuscular strength was examined only one time using the grip strength test according to previous studies^18,55,56^. A grip strength meter was used to assess forelimb strength. Mice were lifted and held by the tail such that their forepaws could grasp a wire grid; they were then pulled back gently until they released the grid. The peak force applied by the forelimbs was recorded in Newton (cN).

### Elevated plus-maze test

Anxiety-like behaviour was examined using the elevated plus-maze according to previous studies^18,36,56^. The apparatus consisted of two opposing open and two opposing closed arms (each 8 × 25 cm), with 30-cm high transparent plastic walls. The arms were constructed from white acrylic plates, and the maze was elevated to a height of 40 cm above the floor. The centre of the apparatus was illuminated at 100 lux. Each mouse was placed in the central square of the maze, facing one of the closed arms, and was allowed to explore freely the four arms for 6 min. The mice were video recorded, and distance travelled (m), number of entries into open arms, ratio of entries into the open arms, and time spent in the open arms (s) were analysed using the ANY-MAZE software.

### Light/dark transition test

Light/dark transition test was examined according to previous studies^18,43^. The apparatus consisted of an acrylic cage (22 × 44 × 40 cm) divided into two sections of equal size by a partition with a door. One chamber had white acrylic walls and was brightly illuminated (200 lx) by lights above the ceiling of the chamber, and the other chamber had black acrylic walls and was dark (50 lx). Both chambers had a white plastic floor. Mice were placed into the dark chamber and allowed to move freely between the two chambers for 6 min with the door open. The distance travelled (m), ratio of distance in the light compartment, total number of transitions, and time spent in the light chamber (s) were analysed using the ANY-MAZE software.

### Open-field test

Exploratory behaviour, anxiety-like behaviour, and general locomotor activity were examined using the open-field test according to previous studies^18,56,57^. Each mouse was placed in the centre of the apparatus consisting of a square area surrounded by white acrylic walls (45 × 45 × 40 cm). The total distance travelled (m) and time spent in the central area (s) were recorded. The central area was defined as the middle 20 × 20 cm area of the field. The test chamber was illuminated at 100 lx. Data were collected over a 30-min period. Data analysis was performed using the ANY-MAZE software.

### Y-maze test

Spatial working memory was assessed using a white acrylic Y-maze apparatus (arm length: 40 cm, arm bottom width: 3 cm, arm upper width: 10 cm, wall height: 12 cm). Mice were placed at the centre of the Y-maze for 6 min. Visual cues (circles, triangles and later an X) were placed around the maze in the testing room and were constant throughout the testing sessions. Mice were tested with no previous exposure or habituation to the maze. A spontaneous alternation was defined as an entry into three different arms on consecutive choices. The percentage of alternation was calculated as the ratio of actual to maximum number of alternations. The total distance travelled (m), number of entries, and number of alternations were recorded and analysed using the ANY-MAZE software.

### Social interaction test

Social interaction test was examined according to previous studies^18,56,58^. The apparatus consisted of a white acrylic rectangular parallelopiped (30 × 60 × 40 cm; Fig. 5a). Each mouse was placed in the box for 6 min and allowed to freely explore for habituation. In the sociability test, an unfamiliar C57BL/6 N male mouse (stranger mouse) that had no previous contact with the subject mouse was placed into one of the transparent plexiglass cages (7.5 × 7.5 × 10.0 cm, which had several holes with a diameter of 1 cm) located at the corners of each lateral compartment (Fig. 5b). The stranger mouse was enclosed in the transparent cage, which allowed nose contact between the bars but prevented fighting. The subject mouse was placed again in the centre of the box and allowed to explore the entire box for a 6-min session. One side of the rectangular area was defined as the stranger area and the other side as the empty area. The total distance travelled (m) and amount of time spent near each cage (around cage; within 20 cm from the corner) during the 6-min sessions were measured. Data were recorded on video and analysed using the ANY-MAZE software.

### Rotarod test

Motor coordination and balance were tested using the rotarod test according to previous studies^59^. This test, which uses an accelerating rotarod (RTR-M5; Melquest, Toyama, Japan), was performed by placing a mouse on rotating drums (3.9-cm diameter) to measure the time that the animal was able to maintain its balance on this rod. The speed of the rotarod was accelerated from 4 to 40 rpm over a 5-min period. The intertrial interval for this test was 20 min. All mice were subjected to the test without any pre-test training.

### Tail suspension test

Depression-like behaviour was examined using the tail suspension test according to previous studies^18,56,60^. Each mouse was suspended by the tail at 60 cm above the floor in a white plastic chamber using adhesive tape placed <1 cm from the tip of the tail. The resultant behaviour was recorded for 6 min. Images were captured via a video camera, and immobility time was measured. In this test, the ‘immobile period’ was defined as the period when the animals stopped struggling for ≥ 1 s. Data acquisition and analysis were performed using the ANY-MAZE software.

### Porsolt forced swim test

The Porsolt forced swim test was also used to examine depression-like behaviour. The apparatus consisted of four Plexiglas cylinders (20 cm [height] × 10 cm [diameter]). The cylinders were filled with water (23 °C) to a depth of 7.5 cm, based on previous studies^61,62^. The mice were placed into the cylinders for 6 min and recorded. As in the tail-suspension test, immobility time was evaluated using the ANY-MAZE software.

### Step-through passive avoidance test

A two-compartment step-through passive avoidance apparatus (MPB-M020; Melquest) was used according to previous studies^63^. The apparatus is divided by a wall with a guillotine door into a bright (9.0 × 18.0 × 14.5 cm) and a dark (18.0 × 18.0 × 14.5 cm) compartment. The bright compartment was illuminated by fluorescent light (200 lx). Mice were placed in the bright compartment and allowed to explore for 20 s, at which point the guillotine door was raised to allow the mice to enter the dark compartment. When the mice entered the dark compartment, the guillotine door was closed and an electrical foot shock (0.5 mA) was delivered for 3 s. Test sessions were performed 24 h after training sessions. The mice were placed in the bright compartment and allowed to explore for 20 s, and then the guillotine door was raised. The latency to enter the dark compartment was recorded for up to 180 s.

### Data analyses

Statistical analyses were performed using the SPSS software (IBM, Armonk, NY, USA). Data were analysed using the two-tailed t-test, one-way ANOVA, or two-way repeated-measures ANOVA. Differences with a p-value <0.05 were regarded as statistically significant. Data are presented as box plots or means ± standard errors.

## Data Availability

All relevant data are presented within the manuscript.

## References

[1] Taylor K, Gordon N, Langley G, Higgins W (2008). Estimates for worldwide laboratory animal use in 2005. Altern. Lab. Anim..

[2] Moberg, G.P. & Mench, J. The Biology of Animal Stress: Basic Principles and Implications for Animal Welfare (Cabi Publishing). (2000).

[3] Meijer MK, Sommer R, Spruijt BM, van Zutphen LF, Baumans V (2007). Influence of environmental enrichment and handling on the acute stress response in individually housed mice. Lab. Anim..

[4] Schmidt MV (2010). A novel chronic social stress paradigm in female mice. Horm. Behav..

[5] Calvo-Torrent A, Brain PF, Martinez M (1999). Effect of predatory stress on sucrose intake and behavior on the plus-maze in male mice. Physiol. Behav..

[6] Archer J (1973). Tests for emotionality in rats and mice: a review. Anim. Behav..

[7] Deacon RM (2006). Assessing nest building in mice. Nat. Protoc..

[8] Claxton AM (2011). The potential of the human–animal relationship as an environmental enrichment for the welfare of zoo-housed animals. Appl. Anim. Behav. Sci..

[9] Krohn TC, Sørensen DB, Ottesen JL, Hansen AK (2006). The effects of individual housing on mice and rats: A review. Anim. Welf..

[10] Benedetti F, Fresi F, Maccioni P, Smeraldi E (2008). Behavioural sensitization to repeated sleep deprivation in a mice model of mania. Behav. Brain Res..

[11] Bohlen M (2014). Experimenter effects on behavioral test scores of eight inbred mouse strains under the influence of ethanol. Behav. Brain Res..

[12] Wahlste, D. Mouse Behavioral Test. Academic Press (2010).

[13] Brigman, J. L., Graybeal, C. & Holmes, A. Predictably irrational: assaying cognitive inflexibility in mouse models of schizophrenia. Front Neurosci. **4** (2010). 10.3389/neuro.01.013.2010 PMC2938983 20859447

[14] Wolf A, Bauer B, Abner EL, Ashkenazy-Frolinger T, Hartz AM (2016). A Comprehensive Behavioral Test Battery to Assess Learning and Memory in 129S6/Tg2576 Mice. PLoS One.

[15] Brooks SP, Dunnett SB (2009). Tests to assess motor phenotype in mice: a user’s guide. Nat. Rev. Neurosci..

[16] Silverman JL, Yang M, Lord C, Crawley JN (2010). Behavioural phenotyping assays for mouse models of autism. Nat. Rev. Neurosci..

[17] Takao K, Miyakawa T (2009). Intrauterine environment-genome interaction and children’s development (4): Brain-behavior phenotypying of genetically-engineered mice using a comprehensive behavioral test battery on research of neuropsychiatric disorders. J. Toxicol. Sci..

[18] Umemura M (2017). Comprehensive Behavioral Analysis of Activating Transcription Factor 5-Deficient Mice. Front. Behav. Neurosci..

[19] Nakajima R (2019). Comprehensive behavioral analysis of heterozygous Syngap1 knockout mice. Neuropsychopharmacol. Rep..

[20] Watanabe, Y. *et al*. Relaxin-3-deficient mice showed slight alteration in anxiety-related behaviour. Front. Behav. Neurosci., **17** (2011). 10.3389/fnbeh.2011.00050 PMC3156976 21887138

[21] Wu, B., Wei, Y., Wang, Y. & Su, T. Gavage of D-Ribose induces Aβ-like deposits, Tau hyperphosphorylation as well as memory loss and anxiety-like behavior in mice. Oncotarget. **6** (2015). 10.18632/oncotarget.6021 PMC4741441 26452037

[22] Wolf, A. *et al*. A Comprehensive Behavioral Test Battery to Assess Learning and Memory in 129S6/Tg2576 Mice. PLOS ONE 25 (2016). 10.1371/journal.pone.0147733 PMC4726499 26808326

[23] Rajesh V, Riju T, Venkatesh S, Babu G (2017). Memory enhancing activity of Lawsonia inermis Linn. leaves against scopolamine induced memory impairment in Swiss albino mice. Orient. Pharm. Exp. Med..

[24] Wahlsten D (2003). Different data from different labs: lessons from studies of gene-environment interaction. J. Neurobiol..

[25] Deacon RM (2006). Housing, husbandry and handling of rodents for behavioral experiments. Nat. Protoc..

[26] Leach MC, Main DCJ (2008). An assessment of laboratory mouse welfare in UK animal units. Anim. Welf..

[27] Mandillo S (2008). Reliability, robustness, and reproducibility in mouse behavioral phenotyping: a cross-laboratory study. Physiol. Genomics.

[28] Hurst JL, West RS (2010). Taming anxiety in laboratory mice. Nat. Methods..

[29] Gouveia K, Hurst JL (2013). Reducing mouse anxiety during handling: effect of experience with handling tunnels. PLoS One.

[30] Gouveia K, Hurst JL (2017). Optimising reliability of mouse performance in behavioural testing: the major role of non-aversive handling. Sci. Rep..

[31] Miller A, Kitson G, Skalkoyannis B, Leach M (2015). The effect of isoflurane anaesthesia and buprenorphine on the mouse grimace scale and behaviour in CBA and DBA/2 mice. Appl. Anim. Behav. Sci..

[32] Nakamura Y, Suzuki K (2018). Tunnel use facilitates handling of ICR mice and decreases experimental variation. J. Vet. Med. Sci..

[33] Patterson ZR, Abizaid A (2013). Stress induced obesity: lessons from rodent models of stress. Front. Neurosci..

[34] Sorge RE (2014). Olfactory Exposure to Males, Including Men, Causes Stress and Related Analgesia in Rodents. Nat. Methods.

[35] Rodgers RJ, Dalvi A (1997). Anxiety, defence and the elevated plus-maze. Neurosci. Biobehav. Rev..

[36] Komada M, Takao K, Miyakawa T (2008). Elevated plus maze for mice. J. Vis. Exp..

[37] Holmes A, Parmigiani S, Ferrari PF, Palanza P, Rodgers RJ (2000). Behavioral profile of wild mice in the elevated plus-maze test for anxiety. Physiol. Behav..

[38] Dohi Eisuke, Choi Eric Y., Rose Indigo V.L., Murata Akiho S., Chow Sharon, Niwa Minae, Kano Shin-ichi (2017). Behavioral Changes in Mice Lacking Interleukin-33. eneuro.

[39] Sakakibara Y, Sekiya M, Saito T, Saido TC, Iijima KM (2018). Cognitive and emotional alterations in App knock-in mouse models of Aβ amyloidosis. BMC Neurosci..

[40] Seibenhener ML, Wooten MC (2015). Use of the Open Field Maze to measure locomotor and anxiety-like behavior in mice. J. Vis. Exp..

[41] Jin S (2018). Anxiety-like behaviour assessments of adolescent rats after repeated maternal separation during early life. Neuroreport.

[42] Bourin M, Hascoët M (2003). The mouse light/dark box test. Eur. J. Pharmacol..

[43] Takao, K. & Miyakawa, T. Light/dark transition test for mice. J Vis Exp **104** (2006). 10.3791/104 PMC2504462 18704188

[44] Kennedy SH (2008). Core symptoms of major depressive disorder: relevance to diagnosis and treatment. Dialogues Clin. Neurosci..

[45] Regier DA, Rae DS, Narrow WE, Kaelber CT, Schatzberg AF (1998). Prevalence of anxiety disorders and their comorbidity with mood and addictive disorders. Br. J. Psychiatry Suppl..

[46] Trivedi MH (2007). Maximizing the adequacy of medication treatment in controlled trials and clinical practice: STAR*D measurement-based care. Neuropsychopharmacology.

[47] Borsini F, Volterra G, Meli A (1986). Does the behavioral “despair” test measure “despair. Physiol. Behavior..

[48] Horii Y (2017). Hierarchy in the home cage affects behaviour and gene expression in group-housed C57BL/6 male mice. Sci. Rep..

[49] Bartolomucci A (2001). Social status in mice: behavioral, endocrine and immune changes are context dependent. Physiol. Behav..

[50] Liu X, Peprah D, Gershenfeld HK (2003). Tail-suspension induced hyperthermia: a new measure of stress reactivity. J. Psychiatr. Res..

[51] Hok V, Poucet B, Duvelle É, Save É, Sargolini F (2016). Spatial cognition in mice and rats: similarities and differences in brain and behavior. Wiley Interdiscip. Rev. Cogn. Sci..

[52] Frick KM, Stillner ET, Berger-Sweeney J (2000). Mice are not little rats: Species differences in a one-day water maze task. NeuroReport: For. Rapid Commun. Neurosci. Res..

[53] Donovan, J. & Brown, P. Care and handling of laboratory mice. Current Protocols in Microbiology A.3N.1–A.3N.18 (2013). 10.1002/9780471729259.mca03ns31 24510294

[54] Akatsu S, Ishikawa C, Takemura K, Ohtani A, Shiga T (2015). Effects of prenatal stress and neonatal handling on anxiety, spatial learning and serotonergic system of male offspring mice. Neurosci. Res..

[55] Shoji H, Takao K, Hattori S, Miyakawa T (2016). Age-related changes in behavior in C57BL/6J mice from young adulthood to middle age. Mol. Brain.

[56] Ueno H (2019). Anti-depressive-like Effect of 2-phenylethanol Inhalation in Mice. Biomed. Pharmacother..

[57] Tamada K (2010). Decreased exploratory activity in a mouse model of 15q duplication syndrome; implications for disturbance of serotonin signalling. PLoS One.

[58] Moy SS (2004). Sociability and Preference for Social Novelty in Five Inbred Strains: An Approach to Assess Autistic-Like Behavior in Mice. Genes. Brain Behav..

[59] Matsuo N (2010). Behavioral Profiles of Three C57BL/6 Substrains. Front. Behav. Neurosci..

[60] Onouchi T (2014). Targeted deletion of the C-terminus of the mouse adenomatous polyposis coli tumor suppressor results in neurologic phenotypes related to schizophrenia. Mol. Brain..

[61] Hagihara H (2016). Circadian Gene circuitry predicts hyperactive behavior in a mood disorder mouse model. Cell Rep..

[62] Ohashi R, Takao K, Miyakawa T, Shiina N (2016). Comprehensive behavioral analysis of RNG105 (Caprin1) heterozygous mice: reduced social interaction and attenuated response to novelty. Sci. Rep..

[63] Shan, Q. *et al*. Purple Sweet Potato Color Ameliorates Cognition Deficits and Attenuates Oxidative Damage and Inflammation in Aging Mouse Brain Induced by D-Galactose. J Biomed Biotechnol, 564737 (2009). 10.1155/2009/564737 PMC2766785 19865488

